# Intracorporeal lithotripsy of salivary stones: in vitro comparison of different methods

**DOI:** 10.1007/s00405-025-09268-1

**Published:** 2025-03-07

**Authors:** Cathrin Schulze, Kruthika Thangavelu, Francesca Gehrt, Robert Schatton, Christian Keil, Hendrik Heers, Nermin H. Abozenah, Boris A. Stuck, Urban Geisthoff

**Affiliations:** 1 Klink für Hals-Nasen-Ohren Heilkunde, Kopf- und Hals-Chirurgie, Universitätsklinikum Marburg, Philipps-Universität Marburg, Marburg, Germany; 2 Klinik für Urologie, Universitätsklinikum Marburg, Philipps-Universität Marburg, Marburg, Germany; 3 HNO Praxis Wülfrath, Wülfrath, Germany; 4 Department of Otolaryngology, Head and Neck Surgery, Philipps-University, Baldingerstrasse, 35043 Marburg, Germany

**Keywords:** Sialolithiasis, Salivary gland stones, Laser lithotripsy, Propulsion, Ho:YAG laser

## Abstract

**Purpose:**

Intracorporeal lithotripsy is a gland-preserving treatment option for sialolithiasis. Laser lithotripsy (LL) and pneumatic lithotripsy (PL) are the only two methods currently approved, the latter being no longer available. Electrokinetic lithotripsy (EKL) is a promising alternative used for the treatment of ureteral stones. The aim of this study is to compare efficacy and therapeutical safety of EKL with LL and PL.

**Methods:**

StoneBreaker^®^ (PL), Lithotron EL 27 Compact (EKL) and Ho:YAG laser Auriga (LL) were assessed using in vitro setups with human salivary stones, casted and tumbled stones. Efficacy was measured by the number of impulses and time taken until fragmentation. Parameters for therapeutical safety were number of impulses until perforation, propulsion, duct widening, number of tears and tear length.

**Results:**

Efficacy of EKL was higher than LL but lower than PL. The fragmentation of casted stones took 01:50 ± 00:28 min with PL, 02:49 ± 00:37 min with EKL and 05:12 ± 00:58 min with LL (Mann–Whitney-U test p < 0.01). LL caused the lowest propulsion (0.0 ± 0 cm, n = 20); the highest propulsion was observed for PL (3.5 ± 0.7 cm, n = 20). In the gelatin setup, LL induced the most extensive damage (damage index: 5.9 ± 2.9, n = 15). LL was the fastest to cause perforation in the parotid duct (1 ± 0 impulses until perforation, n = 10).

**Conclusion:**

Efficacy and safety of EKL are between those of LL and PL. Therefore, clinical testing of EKL seems to be justified.

**Supplementary Information:**

The online version contains supplementary material available at 10.1007/s00405-025-09268-1.

## Introduction

Obstructive sialadenitis is caused in 60–70% of cases by salivary calculi. Prevalence of sialolithiasis has been calculated up to 1% [1, 2]. The initial therapeutical approach in the treatment of sialolithiasis consists of conservative measures like gland massages [2–4]. Patients that present with recurrent and painful gland swelling may require surgical techniques ranging from papillotomy to full gland extirpation [5].

Surgical complications are well reported in the literature and include nerve injuries and duct damage, leading to stenosis and wound infection [6–9]. Interventional sialendoscopy offers a minimally invasive therapeutical approach reducing surgical risks and allows for gland preservation and functional recovery [10]. The combination of different methods in interventional sialendoscopy such as intracorporeal lithotripsy and mechanical retrieval with forceps and baskets has proven to be very successful [2]. Various methods of intraductal fragmentation have been developed including mechanic lithotripsy (ML), pneumatic lithotripsy (PL), electrokinetic lithotripsy (EKL), electrohydraulic lithotripsy (EHL) and laser lithotripsy (LL). LL and PL are approved for the treatment of salivary stones [7, 8, 11–13]. A device for PL has recently been taken off the market, creating the need for alternative treatment options. EKL is approved for ureteral stones [14, 15]. One case report in literature describes its successful use in sialolithiasis [16]. A systematic analysis of efficacy and therapeutical safety of EKL in comparison to the established devices for intracorporeal lithotripsy is still missing. The aim of this study is to compare the in vitro efficacy and therapeutical safety of EKL to the established LL and PL for intracorporeal lithotripsy. We designed a set of in vitro studies that imitate clinical situations to ensure transferability of results and to assess if further clinical testing is recommendable.

## Material and methods

In four in vitro studies we comparatively investigated the efficacy (number of impulses and time until fragmentation) and therapeutical safety (propulsion (cm), a damage index representing duct lengthening, duct widening and tear length and quantity and the number of impulses until perforation) of three intracorporeal lithotripters: pneumatic (PL) StoneBreaker^®^ (Cook Medical), electrokinetic (EKL) Lithotron EL 27 Compact (WALZ Elektronik GmbH; EKL setting C, 950 mJ/pulse, 40 Hz probe: K0,8 diameter: 0.83 mm;) and the Ho:YAG laser (LL) Auriga (StarMedTec GmbH, 1.2 J, 3 Hz and 3.6 W, probe: Laser Fibre LightTrain ™ reusable 230 µm, length 3.0 m). The laser settings were chosen in reference to Koch et al. [13]. The Lithotron EL 27 Compact is a device that allows the application of two physically different techniques, electrohydraulic and electrokinetic lithotripsy, when using different probes [9]. In our studies, only the EKL technique and probes were used.

We tested an artificial stone phantom in comparison to original salivary gland stones. BegoStones are relatively homogenous and have been used before to access devices for ureteroscopic lithotripsy [17–19]. The casted artificial stones phantoms (BegoStones, diameter 5 mm) were handcrafted using Bego powder (BEGO, BEGO GmbH& Co KG Bremen, Germany). The powder to water ratio was 15:4 as it has been used in former studies [18]. The salivary stones were collected in gland surgeries (mean diameter 4.9 mm).

### Fragmentation setup

**Fig. 1:**
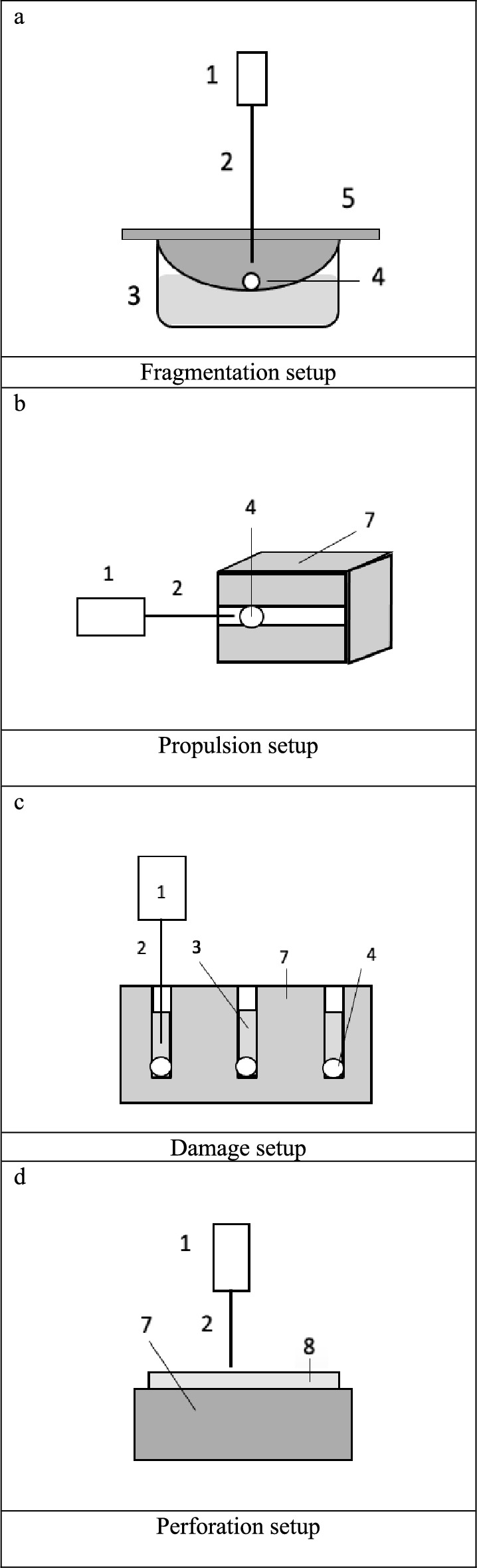
Depiction of the **a** fragmentation, **b** propulsion, **c** damage and **d** perforation setups. Impulses were applied to the stone or duct phantom using different devices for intracorporeal lithotripsy. Measured parameters included number of impulses, application time, propulsion (cm), tear number and length (mm), duct lengthening and widening (mm). 1 device, 2 probe, 3 water, 4 calculus, 5 sieve, 7 gelatin block, 8 duct phantom, e.g. cattle Stensen ducts

Impulses from the handheld devices were directed perpendicularly from the top onto the calculi (diameter 5 mm) as illustrated in Fig. 1A. Stones were positioned on a sieve with a mesh size of 1.5 mm. The same mesh size has been used before in experimental setups and is believed to allow for spontaneous discharge of residual fragments [11, 20, 21]. The stones were submerged in tap water or NaCl solution. By moving the application fiber smoothly across the surface of the stone phantom while maintaining mechanical contact, the stones were ablated. We recorded time and number of impulses. The total application time (t_total_) was defined as the period until all fragments were smaller than 1.5 mm and had therefore fallen through the sieve. In this experimental setup, we tested casted (BegoStones) stones.

### Propulsion setup

For the propulsion setup as well as for the damage setup, we fabricated gelatin blocks (Speisegelatine Qualität 250 Bloom Typ A – Schweinegelatine Nauman, Gelatine und Leim GmbH, Memmingen, Germany). The powder to water ratio was 1:10 as it is widely used in ballistic research to substitute living muscle [22]. When casting the gelatin blocks, we placed rods inside the block that were later removed, creating a duct system in which we positioned and fragmented the stone phantoms (Fig. 1b). In the propulsion setup, we placed the stone phantoms in the opening of the horizontal gelatin duct (diameter 5 mm) and measured the displacement of the stone phantom in response to a single impulse using a ruler. Similar experiments had been conducted before using silicone tubes, pendulum setups or camera systems/video tracking to record the vertical displacement when applying impulses from below [17, 18, 23].

### Damage setup

For visibility reasons, stones were placed in slightly colored water at the end of vertical gelatin ducts and impulses of the devices were applied (Fig. 1c). To measure the induced damage, we recorded the duct widening (mm), duct lengthening (mm), tear number and tear length (mm). Based on experiences from our pre-tests, we decided to combine the recorded results into one parameter by defining a damage index without dimensions that would allow us to comprehensively compare the overall damage induced to the gelatin and duct model. We calculated our damage index by calculating the sum of the parameters as shown below.

$$damage\, index=\frac{duct\, widening}{mm}+ \frac{duct\, lengthening}{mm}+ tear\, number+\frac{tear\, length}{mm}$$

### Perforation setup

Cattle Stensen ducts were cut open lengthwise and positioned on a gelatin block. From above, we applied impulses and measured the number of impulses until the probes perforated the ducts wall and pierced the gelatin block (Fig. 1d).

### Statistical evaluation

Statistical evaluation of the data was carried out using SPSS Version 28.0.0. We tested our data for normal distribution with the Shapiro–Wilk normality-test and calculated the power using the software G*Power 3.1 for Mac. To test the significance of differences of medians between the device combinations EKL and PL and EKL and LL, we conducted Mann–Whitney-U tests.

Additionally, we conducted noninferiority studies and in case of shown noninferiority we switched to potentially showing superiority of the new device EKL. The margins of the equivalence interval were set to ± 20% of the mean in analogy to Cohen’s d, where d = 0.20 is widely considered to be a small effect [24]. The linear regression was calculated using the program R. All data is provided as mean ± SD, time was always measured in minutes.

## Results

### Efficacy

The results of linear regression analysis revealed a significant association between the fragmentation time and the number of impulses (p < 0.05), independently from device, stone phantom or experimental setup.

**Fig. 2:**
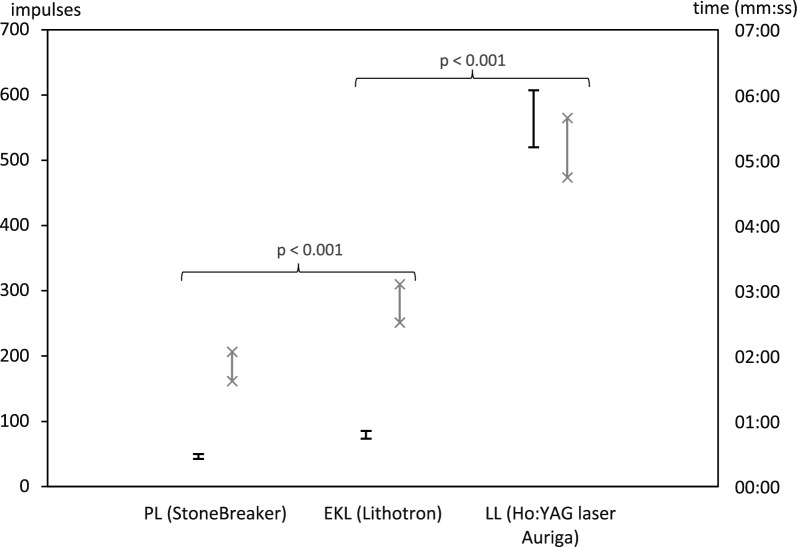
Efficacy of PL, EKL and LL. The 95% confidence interval of the number of impulses until fragmentation on the sieve is depicted as horizontal bars, 95% confidence interval of the time until perforation as crosses. Mann–Whitney-U tests were performed to test if the differences of medians of PL, EKL and LL were statistically significant

Fragmentation by EKL was less efficient than by the pneumatic and more efficient than by the laser lithotripter (Fig. 2). Complete fragmentation of pre-watered BegoStones on the sieve was achieved by PL with 46.1 ± 8.1 (mean ± SD) impulses, while 79.4 ± 12.8 with the EKL and considerably more impulses with the LL (563.6 ± 93.6 impulses) were necessary. Mean duration of fragmentation was 01:50 ± 00:28 min for PL, 02:49 ± 00:37 min for EKL and 05:12 ± 00:58 min for LL. The differences of medians were statistically significant for the parameters impulses and time when comparing EKL and PL as well as EKL and LL (p < 0.001). The effect size Cohen’s d was 3.105, 1.757, – 7.266 and – 2.927, respectively and thereby large in all combinations (d ≥ 0.8).

As PL was more efficient than EKL, noninferiority of the EKL could not be shown, while noninferiority and superiority of the EKL over the LL was clearly established (impulses: C_u_ = − 440.246, ε_1_ = − 112.7 and ε_2_ = 112.7 $\to$ C_u_ < ε_1_; ε_2_). This data is depicted in Tables 1 and 2.

**Table 1:** Fragmentation of pre-watered BegoStones on the sieve-descriptive statistics

Device	Parameter	Mean	SD	n	Min	Max	Median	95% confidence interval of the mean	
								C_l_	C_u_
StoneBreaker (PL)	Impulses^a^	46.1	8.1	20	31	62	45.5	42.3	49.9
	Time	01:50	00:28	20	00:48	02:44	01:54	01:37	02:04
EKL K0,8	Impulses	79.4	12.8	20	60	110	76.5	73.4	85.3
	Time	02:49	00:37	20	01:42	03:58	02:43	02:31	03:06
Ho:YAG laser (LL)	Impulses	563.6	93.4	20	410	848	567.0	519.9	607.3
	Time	05:12	00:58	20	03:50	08:33	05:10	04:44	05:39

^a^Number of impulses and application time until complete fragmentation in the sieve setup of the devices PL, EKL and LL (time (min.:s)

**Table 2:** Fragmentation of pre-watered BegoStones on the sieve – Noninferiority study (with extension to superiority)

Device	Parameter	Differences compared to the EKL^a^										
		Man-Whitney U test		Effect size	Power	Mean difference	95% Confidence interval of the difference		Equivalence margins^b^		Non-inferiority of EKL shown^c^	Superiority of EKL shown
		U	p	Cohen’s d			C_l_	C_u_	ε_1_ (− 20%)	ε_2_ (+ 20%)		
StoneBreaker (PL)	Impulses	9.820	< 0.001	3.105	1.000	33.300	26.394	40.206	− 9.22	9.22	No	–
	Time	− 5.557	< 0.001	1.757	1.000	00:58	00:37	01:19	− 00:22	00:22	No	–
Ho:YAG laser (LL)	Impulses	− 22.977	< 0.001	− 7.266	1.000	− 484.250	− 528.254	− 440.246	− 112.7	112.7	Yes	Yes
	Time	− 9.255	< 0.001	− 2.927	1.000	− 02:22	− 02:53	− 01:51	− 01:02	01:02	Yes	Yes

^a^Results of the noninferiority studies are expressed as a confidence interval for the difference between the test treatment (EKL) and control (PL, LL)

^b^Equivalence margins were chosen according to Cohen’s small effect size 0.20 and therefore calculated by mean of the standard devices PL or LL ± 20% [24]

^c^Noninferiority regarding the device’s efficacy was shown if C_u_ ≤ ε_2_, superiority was shown, when C_u_ < ε_1_. C_u_ = upper margin of the 95% CI

### Therapeutical safety

**Fig. 3:**
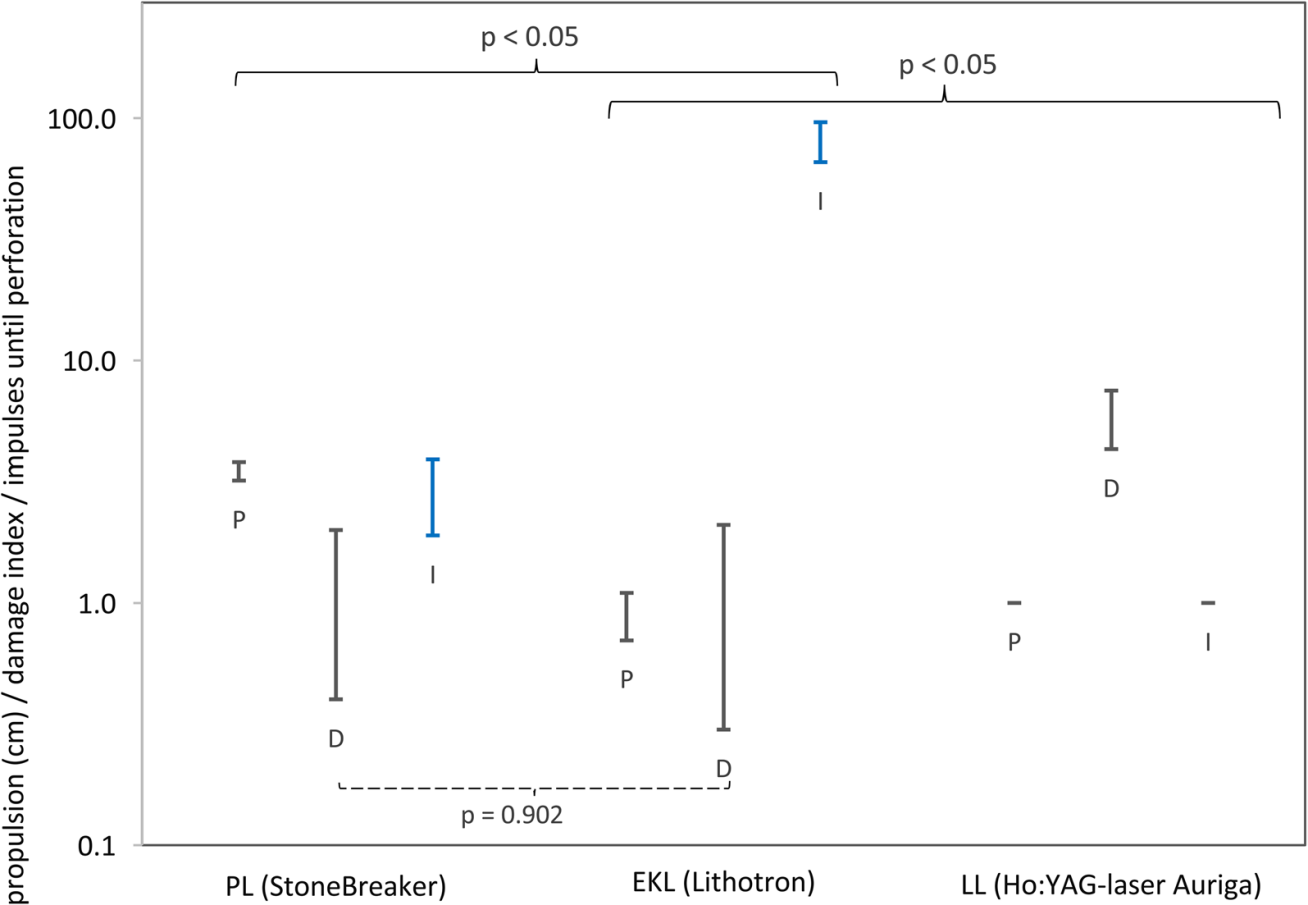
Therapeutical safety parameters propulsion (P), damage index (D) and number of impulses (I) until perforation obtained with StoneBreaker, EKL and LL. The ordinate is scaled logarithmically. Illustrated are the margins of the 95% CI of the means. Higher propulsion and higher damage index indicate lower therapeutical safety while a larger number of impulses until perforation of the duct phantom indicates a higher therapeutical safety. The Mann–Whitney-U tests showed that all differences of medians were statistically significant (p < 0.05), except for StoneBreaker: EKL (damage index p = 0.902 as indicated by the dotted parenthesis)

Results of the safety experiments are summarized in Tables 3 and 4 and depicted in Fig. 3. Propulsion of the casted stone phantoms (BegoStones) was lowest with the LL (M = 0.0, SD = 0.0 cm) and highest with the PL (M = 3.5, SD = 0.7 cm). The EKL’s propulsion was in between these two devices (M = 0.9, SD = 0.5 cm). The difference of medians between PL, respectively LL and EKL (PL: U = 117.500, p = 0.024; LL: U = 0.000 p = 0.001) was statistically significant. In both comparisons, the effect size was large (Cohen’s d = − 4.499 respectively d = 2.681). EKL was shown to be both noninferior and also superior (C_u_ = − 2.2496 < ε_1_ = − 0.7; ε_2_ = 0.7) to PL.

**Table 3:** Descriptive statistics of the therapeutical safety of PL, EKL and LL in the propulsion, damage and perforation setup

Device	Parameter	Mean	SD	n	Min	Max	Median	95% confidence interval	
								C_l_	C_u_
StoneBreaker (PL)	Propulsion^a^	3.5	0.7	20	3.0	5.0	3.0	3.2	3.8
	Damage^b^	1.2	1.4	15	0.0	4.0	1.0	0.4	2.0
	Perforation^c^	2.7	0.8	10	2.0	4.0	2.5	2.1	3.3
EKL K0,8	Propulsion	0.9	0.5	20	0.5	2.0	1.0	0.7	1.1
	Damage	1.2	1.6	15	0.0	4.0	0.0	0.3	2.1
	Perforation	81.0	25.9	10	23.0	110.0	87.0	62.5	99.5
Ho:YAG laser (LL)	Propulsion	0.0	0.0	20	0.0	0.1	0.0	0.0	0.0
	Damage	5.9	2.9	15	2.0	10.0	7.0	4.3	7.5
	Perforation	1.0	0.0	10	1.0	1.0	1.0	1.0	1.0

^a^Propulsion in cm

^b^The damage was quantified by a damage index, a parameter without dimensions calculated by adding number of tears, tear length, duct widening and lengthening

^c^Number of impulses until perforation

**Table 4:** Noninferiority study (with extension to superiority)

Device	Parameter	Differences compared to the EKL										
		Mann–Whitney U test		Effect size	Power	Mean difference	95% confidence interval of the difference		Equivalence margins		Non-inferiority of EKL shown	Superiority of EKL shown
		U	p	Cohen’s d			C_l_	C_u_	ε_1_ (− 20%)	ε_2_ (+ 20%)		
StoneBreaker (PL)	Propulsion^a^	117.500	0.024	− 4.499	1.000	− 2.6250	− 3.0004	− 2.2496	− 0.7	0.7	Yes	Yes
	Damage	109.500	0.902	0.000	0.522	0.00000	− 1.12018	1.12018	− 0.24	0.24	No	–
	Perforation^b^	0.000	< 0.001	4.271	0.789	78.300	59.758	95.525	− 0.54	0.54	Yes	Yes
Ho:YAG laser (LL)	Propulsion	0.000	< 0.001	2.681	1.000	0.8650	0.6516	1.0784	0	0	No	–
	Damage	18.500	< 0.001	− 2.340	0.522	− 4.73333	− 6.50797	− 2.95870	− 1.18	1.18	Yes	Yes
	Perforation	0.000	< 0.001	4.366	0.789	80.000	61.462	98.538	− 0.2	0.2	Yes	Yes

^a^For the setups propulsion and damage, the same decision rules apply for the noninferiority study as in the efficacy testing

^b^In the perforation setup, more impulses are associated with higher therapeutical safety. Noninferiority regarding the device’s therapeutical safety was therefore shown, if C_l_ ≥ ε_1_, and superiority, when C_l_ > ε_2_. C_l_ = lower margin of the 95% CI

**Fig. 4:**
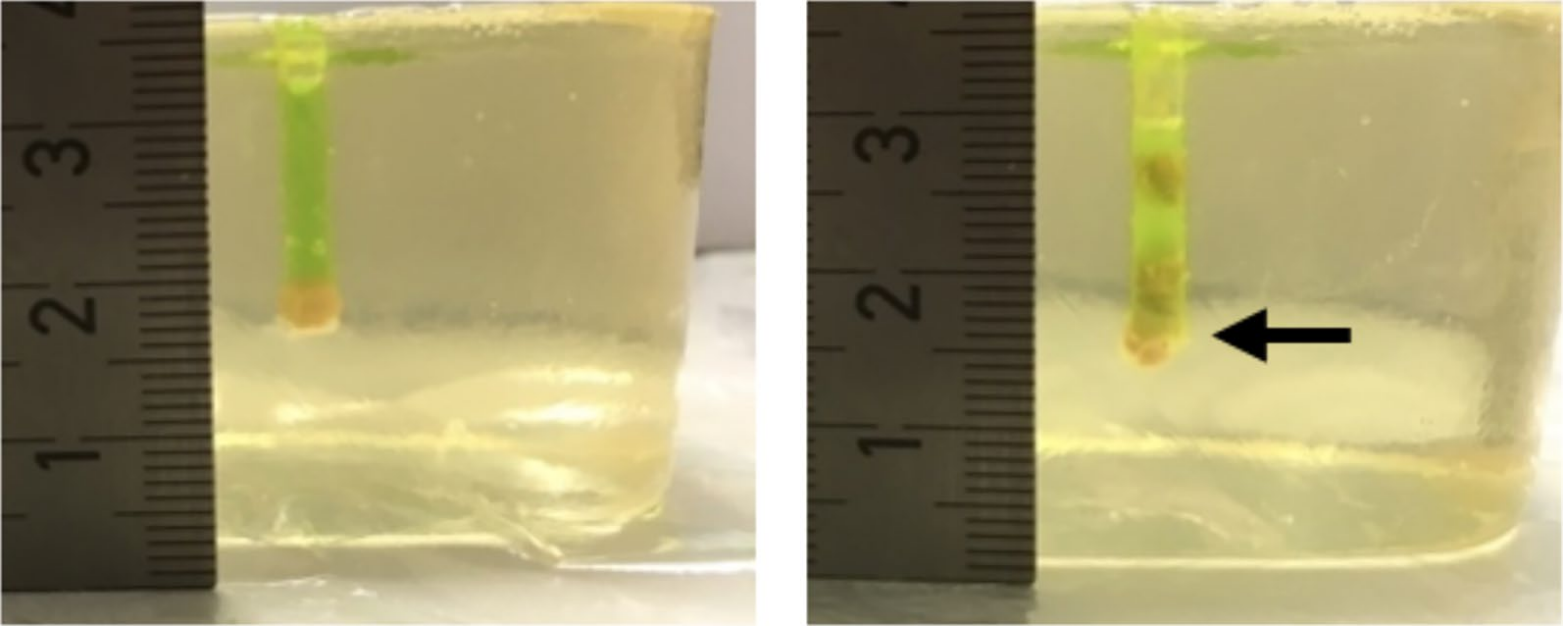
Depiction of the damage setup. BegoStones were placed in vertical ducts of gelatin blocks. On the left side before and on the right side after the application of impulses

The application of impulses to the casted stones inside the gelatin block caused more damage with the LL (M = 5.9, SD = 2.9) than compared to all other devices (example: Fig. 4). PL (M = 1.2 SD = 1.4) and EKL (M = 1.2, SD = 1.6) were recorded to show much lower damage indices. EKL induced significantly less damage (U = 18,500, p < 0.001) than LL and was superior (C_u_ = − 2.95870 < ε_1_ = − 1.18; ε_2_ = 1.18) to the LL. The Mann–Whitney-U test revealed no significant difference between the application of impulses of PL and EKL (U = 109.500, p = 0.902).

Impulses of the LL were the quickest to cause perforation in the duct phantoms (M = 1.0 SD = 0.0 impulses). The experiment was repeated 10 times for each device. With the PL 2.7 ± 0.8 impulses could be applied until perforation of the duct phantoms. EKL caused perforation after significantly (p < 0.001) more impulses (M = 81.0, SD = 5.9 impulses) and was superior to both PL (C_l_ = 59.758 > ε_1_ = − 0.54, ε_2_ = 0.54) and LL (C_l_ = 61.462 > ε_1_ = − 0.2, ε_2_ = 0.2) – the effect size was large (Cohen’s d = 4.271 respectively d = 4.366).

### Salivary stone test series

Comparison of BegoStone test series to salivary stone test series as an assessment of the potential transferability into clinical settings.

In Table 5 the results of the efficacy tests are summarized for the BegoStone and salivary stone test series. The mean values and standard deviation are illustrated as obtained in the setup fragmentation on sieve until complete fragmentation. In both test series, the number of impulses needed to fragment the stone phantoms was much lower with PL (salivary stones: 72.0 ± 14.6 impulses, BegoStones: 46.1 ± 8.1 impulses) than compared to LL (salivary stones 459.6 ± 349.4 impulses, BegoStones: 563.6 ± 93.4 impulses). With the device EKL K0,8, less impulses were required to fragment salivary stones (64.0 ± 25.2 impulses) than to fragment BegoStones (79.4 ± 12.8 impulses), while PL required more impulses to fragment salivary stones (72.0 ± 14.6 impulses) than BegoStones (46.1 ± 8.1 impulses). EKL (64.0 ± 25.2 impulses) needed even less impulses than PL (72.0 ± 14.6 impulses) to fragment salivary stones (n = 5 and SD relatively high with EKL: SD = 25.2 and PL: SD = 14.6 impulses).

**Table 5:** Comparison of BegoStone and salivary stone test series—descriptive statistics

Efficacy	Device	BegoStones	n	Salivary stones	n
Fragmentation setup^a^	PL	46.1 ± 8.1 01:50 ± 00:28	20	72.0 ± 14.6 02:42 ± 00:36	5
	EKL	79.4 ± 12,8 02:49 ± 00:37	20	64.0 ± 25.2 02:59 ± 01:31	5
	LL	563.6 ± 93.4 05:12 ± 00:58	20	459.6 ± 349.4 02:52 ± 01:42	5

Therapeutical safety	Device	BegoStones	n	Salivary stones	n
Damage setup^b^	PL	1.2 ± 1.4	15	0.4 ± 0.5	5
	EKL	1.2 ± 1.6	15	0.6 ± 0.5	5
	LL	5.9 ± 2.9	15	3.2 ± 0.8	5

^a^Results of StoneBreaker (PL), EKL and Ho:YAG laser (LL), number of impulses (mean ± SD and time (min.:ss ± SD))

^b^Damage index ± SD

The duration times in the salivary stone test series were similar with all three devices (PL: 02:42, EKL: 02:50, LL: 02:52 min) while in the BegoStone test series, duration times differed markedly (PL: 01:50, EKL 02:40, LL: 05:12 min). Considering the number of impulses and duration, EKL performed with higher efficacy in the salivary stone test series than in the BegoStone test series. Both test series showed high damage indices for LL (5.9 ± 2.9 and 3.2 ± 0.8) and low indices for PL (1.2 ± 1.4 and 0.4 ± 0.5) and EKL (1.2 ± 1.6 and 0.6 ± 0.5).

## Discussion

Intracorporeal lithotripsy is an important option for the treatment of sialolithiasis. Two options are currently favored in the treatment of sialolithiasis: a Ho:YAG laser (LL) and a pneumatic lithotripter (PL) [9, 11, 25, 26]. The Ho:YAG laser is widely used and available in urology. Because of this reason it also became the predominately used laser for sialolithiasis [26]. We chose LL and PL as standards for comparative measurements with EKL.

### Fragmentation

Fragmentation is a measure for efficacy [27]. Published in vitro studies offer a variety of fragmentation setups, using different stone phantom sizes and assessing parameters such as dusting ratio (mg/s), weight loss (mg/pulse), energy used, number of impulses and duration—limiting comparability of devices across different studies [17, 18, 28, 29]. Additional data are given in the Online Resource 1. Literature is lacking systematic quantitative in vitro comparisons of fragmentations rates of different devices for intracorporeal lithotripsy.

Our results of the BegoStone fragmentation on the sieve for PL and LL are best comparable to a study published by Wang et. al in 2012: While Wang et al. used 1 cm sized BegoStones and sieve holes of 4 mm, we used 5 mm sized BegoStones and sieve holes of 1.5 mm. However, Wang reported a lower number of impulses for PL (29.0 ± 3.7 impulses) but similar application time (02:02 ± 00:56 min.:s) than we did (PL: 46.1 ± 8.1 impulses, 01:50 ± 00:28 min.:s). Application times with LL were similar in both studies (Wang: 05:04 ± 01:40 min.:s, our study: 05:12 ± 00:58 min.:s). The relative similarity of results – despite the different stone sizes – is a hint for the reproducibility of our experiments. The comparison to other studies e.g. by Siedek, Eisel and Andreeva et al., who compared different lasers and their settings or used greatly differing models (as listed in the online resource) is not possible in such a direct way, but their results are within the range of experiences we made [18, 28–30].

It is possible to apply bursts with the EKL as well as with the Ho:YAG laser but not with the pneumatic lithotripter that we used. By using bursts, more impulses can be applied per time. In our study, we counted and applied single impulses only. That indicates that EKL and the Ho:YAG laser might perform more efficiently in clinical settings than in our in vitro-results.

Interestingly, EKL needed less impulses (64.0 ± 25.2 impulses) to fragment salivary stones than PL (72.0 ±  14.6 impulses). This might be a hint that EKL might perform even better in a clinical setting than in our study. It has to be considered, however, that salivary stones vary in size and composition.

In recent years, a new Thulium fiber laser emerged on the urology market for which a higher stone-free rate was reported than compared to the classic Ho:YAG laser. As laser setting, we chose a fragmentation mode with high energy and low frequency. A different choice of laser or its settings, e.g. a higher frequency, might have led to differing efficacy results than presented here for LL [31].

### Correspondence of in vitro with clinical experiences

A comparison of different devices in clinical settings has not been published, yet. The existing literature allows for only a very limited comparison of devices due to different settings, heterogeneity of salivary stones and patients. Comprehensive data published on success rates and treatment times for PL, EKL, EHL and LL in in vivo studies can be reviewed in our Online Resource 2. Several authors reported clinical experiences using PL and LL [11, 12, 25, 26]. In summary, the results (e.g. treatment times) from our in vitro experiments (PL: 01:50 min and 46.0 impulses, LL 05:12 min) are hardly comparable to clinical experiences (Koch 2022: PL: 50.96 min and 366.5 impulses, Koch 2019 LL: 106 min) but the relative difference between duration time with PL and LL (meaning that PL treatment takes considerably less time than LL treatment) that we found in our in vitro experiments is also apparent in clinical treatments [12, 13].

EKL has been established as a clinical method in urologic lithiasis [14, 15]. A case report describes the successful use of “electrokinetic” lithotripsy in a patient with a parotid stone; the treatment took about 45 min which is similar to the times reported for PL and lower than LL which can also be supported by the results of this study [16]. This can be interpreted as indication that our study results correspond with clinical experiences.

### Safety

When researches first studied the applicability of intracorporeal lithotripters that were already used in the field of urology as treatment option for sialolithiasis, concerns were raised regarding their safety. PL and EHL’s safety was discussed controversially in the literature, for example by Iro et al. and Zenk et al. who deemed their clinical use not suitable [32–35]. Since then, numerous reports have been published, reporting the safe and effective clinical use of PL [11, 12, 25, 36]. Reported side effects include propulsion, tissue damage and perforation, resulting in duct destruction, stenosis, gland infection, necessitating prolonged and repeated antibiotic treatment and endoscopic and surgical therapy up to gland extirpation [2, 11, 12, 28].

This undermines the importance of in vitro safety testings of new devices for intracorporeal lithotripsy, such as the EKL. Existing in vitro experiments focus on ablation rates or propulsion but do not take the induced damage to surrounding tissue into account [17, 18, 23, 28, 29, 37]. Therefore, we developed new in vitro setups to measure duct widening, elongation, tear number and tear length. By considering the combination of ablation rate, propulsion, tissue damage and perforation instead of looking at only one parameter, we expect a more realistic and more valid depiction of the suitability for the clinical use.

### Propulsion

Propulsion of stones while undergoing intracorporeal treatment can lead to dislocation, the retropulsion of sharp-edged stone fragments has been reported to cause duct perforation, especially when using PL [32, 33]. Losing and having to chase the stone under endoscopic control causes a prolonged treatment duration and incomplete stone-clearance can obstruct the duct, impeding salivary outflow and leading to recurring swelling of the gland [2, 18, 23].

Overall, our study results regarding propulsion were roughly in accordance with those previously published and showed that LL causes lower propulsion effects than other devices for intracorporeal lithotripsy [17, 23]. Previously published propulsion rates are shown in the Online Resource 3. Marguet et al. compared stone retropulsion and fragmentation of BegoStones and plaster of Paris phantoms in two lasers and a pneumatic lithotripter [17]. They found that propulsion caused by lasers was significantly lower than by the pneumatic lithotripter. Rühm et al. investigated retropulsion effects of a mechanical lithotripter (LithoBreaker), PL (StoneBreaker) and LL (Ho:YAG laser) using steel spheres [23]. They also described a significantly lower retropulsion for LL than for PL and ML. The overall accordance of our results in the propulsion test series with those from our literature research is an indication for the validity of our study results. However, comparability is limited, due to differing setups. Marguet et al. used cylindrical stone phantoms, we used spherical stone phantoms. We manufactured a gelatin tube to measure propulsion, the authors Rühm, Andreeva and Marguet used glass or plastic tubes in their studies [17, 23, 29].

### Tissue damage

In search of optimal in vitro settings, we generally preferred gelatin blocks over human or animal tissue, because they need more resources. With animal or human tissue, only a low number of tests has been performed (e.g. Koch 1995: n = 4 human submandibular glands) [38]. Additionally, ethical considerations ask for replacements [39, 40]. The availability of tissue from human cadavers is limited and fixation methods can change tissue properties. Gelatin blocks have been used in ballistic experiments because of their similarity to human soft tissue [22, 41]. Additionally, gelatin blocks are homogenous and of constant quality.

During our preliminary pretests we experienced difficulties in describing the tissue damage sufficiently and developed a damage index by combining four parameters, which correlated with our subjective impression of the damage observed in the tissue. As this is a new model, the comparison to existing literature is limited, similar in vitro studies have not been conducted.

In our study, the damage indices obtained with PL and EKL were much lower than with LL. LL caused the most extensive damage (approximately six times higher than PL and EKL). We speculate that this is based on the shortcoming of the simple gelatin ducts to withstand thermal and mechanical effects. Human tissue reacts to laser energy with coagulation, while gelatin starts to melt.

In 2019 and 2022, Koch and colleagues reported low complications rates for the treatments of patients with salivary gland stones with PL and LL. After treatment with LL, the authors reported no side effects or complications, they implanted stents in 4/12 cases, in 54/54 cases, respectively (in the Erlangen and McKay research center). After PL treatment, Koch and colleagues implanted stents in 23/77 cases and reported complications (duct perforation, papillary stenosis and ducts stenosis) in 3 cases [12, 13]. It is unclear, whether the treatments would have led to stenosis without the implantation of a stent. Rahman (2004) and colleagues treated 55 patients with ureteric stones with EKL and observed no perforation or stricture [15].

Assuming clinical transferability of our tissue damage setup to clinical experiences, we expect, when clinically testing EKL, to have similar low complication rates as PL and LL. This assumption however, needs to be assessed in further clinical testing.

### Perforation

Perforation can contribute to inflammatory processes and the development of stenoses which is a complication that has been reported for PL as well as for LL [7, 12, 28]. In an in vitro study, Siedek and colleagues applied single laser impulses to sections of human submandibular ducts and histologically examined depth and width of the induced tissue damage under a light microscope [28]. The application of direct laser light resulted in perforation, reaching up to the muscularis propria. In our study, similar to the experience of Siedek et al., one single impulse by LL was enough to perforate the duct wall, while it withstood more impulses of EKL (81 impulses) and PL (2.7 impulses). In order to gain reliable data on the capacity of EKL causing stenosis of the treated salivary duct, further clinical testing is necessary.

### Study limitations

Several limitations of our study need to be acknowledged: The number of studies comparing different devices for intracorporeal lithotripsy is low. The heterogeneity and incompleteness of existing studies makes the assessment of a new device like EKL difficult. Reproducibility and consistency of our newly developed methods (tissue damage and perforation, damage index) have not been established. Heterogeneity in the literature exists also in the absence of a generally accepted stone phantom: BegoStones, COM stones and plaster of Paris stones are used, limiting transferability among studies [17, 29, 42].

To validate the transferability of results, authors in the field of intracorporeal lithotripsy have considered the parameters biological relevance and clinical outcome correlation [7, 11, 12, 22, 25]. To ensure biological relevance, we matched our in-vivo experiments as closely to clinical conditions as possible by using original salivary stones. As expected, the results in the salivary stone and BegoStone test series differed considerably but were overall consistent, which indicates consistency and transferability. But, evidence of this is highly limited due to the relatively small numbers in the salivary stone test series (n = 5) and high standard deviation reaching from about 25–75% from the initial value (M).

Authors have mainly used clinical outcome correlation to validate the transferability of their in vitro experiments to clinical experiments, which is what we did in this study, as well. The preclinical nature of our study and use of non-validated setups make it difficult to predict the transferability of results. Presuming the transferability of our results might lead to confounding bias.

Another limitation lies in the statistical evaluation: The appropriate choice of equivalence margins is difficult but crucial in the evaluation of noninferiority studies [43]. Especially later adaptation based on the results of the trial is subject to bias [44, 45]. According to Cohen’s widely accepted and cited small effect size 0.20, we set our margins to the mean of the standard device ± 20% [24]. Switching from noninferiority to superiority is discussed controversly in literature [44].

## Conclusion

To our knowledge this is the first study comparing fragmentation rates, propulsion effects, induced damage to surrounding tissue and perforation of three different lithotripsy methods (pneumatic, electrokinetic and laser lithotripsy) in reproducible experimental setups. Our observations regarding PL and LL resemble those made in other in vitro experiments. The newly developed experiments on damage and perforation should be correlated with clinical data to assess their relevance. According to the results of this study, EKL can be considered as a potential new method for the intracorporeal treatment of salivary stones; EKL’s results lay in the same range of well-established methods and allowed effective fragmentation while causing low side effects. Taking the study conditions into account, we deem the clinical testing of EKL in the treatment of sialolithiasis justifiable.

Prospective studies seem recommendable to investigate long term effects of treatment with different devices for intracorporeal lithotripsy devices. Endpoints of particular interest could be symptom/stone-free rates and the assessment of complications such as disruption of the ducts (e.g. stenosis due to duct damage induced by intracorporeal lithotripsy) and recurrent sialadenitis in follow-up examinations.

## Electronic supplementary material

Below is the link to the electronic supplementary material.

Supplementary file1 (PDF 212 kb)

Supplementary file2 (PDF 224 kb)

Supplementary file3 (PDF 205 kb)

Supplementary file4 (SAV 1 kb)

Supplementary file5 (SAV 0 kb)

Supplementary file6 (SAV 1 kb)

Supplementary file7 (SAV 0 kb)

## Data Availability

Available, if requested.
