# Supplementary material for: Intracorporeal lithotripsy of salivary stones: in vitro comparison of different methods
Source: Eur Arch Otorhinolaryngol. 2025 Mar 7;282(6):3233–44. doi: 10.1007/s00405-025-09268-1 (PMC12122608; doi:10.1007/s00405-025-09268-1)
Supplement: Supplementary file 1 — Supplementary file1 (PDF 212 kb) [file 405_2025_9268_MOESM1_ESM.pdf]

Supplementary Information

Intracorporeal lithotripsy of salivary stones: in vitro comparison of different methods

European Archives of Oto-Rhino-Laryngology

Schulze Cathrin[1], Thangavelu Kruthika[1], Gehrt Francesca[1], Schatton Robert[3], Keil Christian[2], Heers Hendrik[2], Abozenah Nermin H.[1], Stuck Boris A.[1], Geisthoff Urban[1]

[1] Klink für Hals-Nasen-Ohren Heilkunde, Kopf- und Hals-Chirurgie, Universitätsklinikum Marburg
[2] Klinik für Urologie Universitätsklinikum Marburg, Philipps-Universität Marburg,
[3] HNO Praxis Wülfrath

Address for correspondence:
Cathrin Schulze: Univ.-HNO-Klinik, Baldingerstrasse, 35043 Marburg, Tel. 06421 58-66488, Fax -66367,
Email: (cathrinschulze@gmx.de)

Online Resource 1

Table 1: Published in vitro studies regarding fragmentation rates and time

Author	Year	Setting	Stone phantom size	Device	Fragmentation impulses or energy used M ± SD	Fragmentation stone clearance time (s)
Iro	1993	Extirpated human submandibular glands	salivary stones	EHL	Stone clearance 53/58 (91%). In 5 cases (9%) no effects were seen	
Vorreuther	1998	Under water fragmentation and measurement of displacement	chalk hard clay cubes 10 x 10 mm	Lithoclast (pneumatic)	Weight loss 24 ± 4 mg/pulse for 20 pulses	
				EKL Combilith	Weight loss 25 ± 3 mg/pulse for 20 pulses	
Marguet	2005	Underwater fragmentation in vertical syringe with 2 mm hole	plaster of Paris stones -	Nd:YAG Laser	at 300 J mean % weight loss: 44,85%, at 400 J: 86,8%	
				Ho:YAG-Laser	Total energy J of 300: mean % weight loss: 3,26%, at 480 J 7,1%	
Siedek	2008	Fragmentation on sieve 1x3 mm mesh	salivary stones	Nd:YAG Laser	107,0 ± 84,5 impulses	
				Ho:YAG-Laser	175,9 ± 120,8 impulses	
Wang	2012	Under water fragmentation on 4 mm sieve 10 mm	spherical BegoStones 1 cm	LithoBreaker	430 ± 97 impulses	484 ± 79 s
				StoneBreaker	29 ± 3,7 impulses	122 ± 56 s
		Ureteroscopic stone fragmentation in horizontal tube		LithoBreaker	178,2 ± 54,1 impulses	97,4 ± 26,7 s
				StoneBreaker	33,2 ± 19,8 impulses	142,6 ± 121,3 s
				Ho:YAG-Laser	1,84 ± 0,3 kJ	304,4 ± 99,9 s
Martov	2014	Fragmentation on sieve 2 mm mesh	cuboid BegoStones 100/256/320 mm^3	EHL	Stone clearance 83% 2 failures in 12 tests	
Schrötzlmair	2015	Fragmentation on mesh sized 1,5 mm	Salivary stones	Ho:YAG-Laser	Fragmentation rate 1J/pulse: 0,41 ± 0,33 mg/pulse	
Hoffmann	2016	Fragmentation in a silicone duct model	salivary stones	StoneBreaker	98 impulses	32 minutes
Eisel	2018	Underwater setup mesh size 2,3 mm	BegoStones cubical 5 mm spherical 6 mm	Ho:YAG-Laser	Dusting ratio 60% at 1J/pulse, 0,3 ms, 10 Hz 73% at 1J/pulse, 1,2 ms, 10 Hz	
Andreeva	2020	Underwater setup with perforated cuvette: 3 mm or 1 mm diameter Horizontal tube	COM stones 8 – 10 mm Uric acid stones 6 – 8 mm	Thulium fiber laser	Dusting < 1mm COM stones 0,2 J, 40 Hz, 8W: 0,6 ± 0,2 mg/s	228 s
					Dusting (<1 mm), uric acid stones 0,2 J, 40 Hz, 8 W: 1,1 ± 0,2 mg/s	294 s
				Ho:YAG-Laser	Dusting < 1mm COM stones 0,2 J, 40 Hz, 8W: 0,2 ± 0,1 mg/s	1015 s
					Dusting (<1 mm), uric acid stones 0,2 J, 40 Hz, 8 W: 0,6 ± 0,1 mg/s	short pulse 495 s

Table 2: Selected published in vitro studies regarding fragmentation rates and time – those more suitable to compare to our study

**Author**	**Year**	**Setting**	**Stone phantom size**	**Device**	**Fragmentation**	
					**impulses or energy used M ± SD**	**stone clearance time**
Siedek	2008	Fragmentation on sieve 1x3 mm mesh	salivary stones	Nd:YAG Laser	107,0 ± 84,5 impulses	
				Ho:YAG-Laser	175,9 ± 120,8 impulses	
Wang	2012	Under water fragmentation on 4 mm sieve 10 mm	spherical BegoStones 1 cm	LithoBreaker	430 ± 97 impulses	484 ± 79 s<br>08:04 ± 01:19 min.:s
				StoneBreaker	29 ± 3,7 impulses	122 ± 56 s<br>02:02 ± 00:56 min.:s
		Ureteroscopic stone fragmentation in horizontal tube		LithoBreaker	178,2 ± 54,1 impulses	97,4 ± 26,7 s<br>01:37 ± 00:27 min.:s
				StoneBreaker	33,2 ± 19,8 impulses	142,6 ± 121,3 s<br>02:23 ± 02:01 min.:s
				Ho:YAG-Laser	1,84 ± 0,3 kJ	304,4 ± 99,9 s<br>05:04 ± 01:40 min.:s
Hoffmann	2016	Fragmentation in a silicone duct model	salivary stones	StoneBreaker	98 impulses	32 minutes
Andreeva	2020	Underwater setup with perforated cuvette: 3 mm or 1 mm diameter<br>Horizontal tube	COM stones<br>8 – 10 mm<br>Uric acid stones<br>6 – 8 mm	Thulium fiber laser	Dusting < 1mm COM stones<br>0,2 J, 40 Hz, 8W: 0,6 ± 0,2 mg/s	228 s
					Dusting (<1 mm), uric acid stones<br>0,2 J, 40 Hz, 8 W: 1,1 ± 0,2 mg/s	294 s
				Ho:YAG-Laser	Dusting < 1mm COM stones<br>0,2 J, 40 Hz, 8W: 0,2 ± 0,1 mg/s	1015 s
					Dusting (<1 mm), uric acid stones<br>0,2 J, 40 Hz, 8 W: 0,6 ± 0,1 mg/s	short pulse<br>495 s
This study		Underwater fragmentation on sieve mesh size 1,5 mm	BegoStone 5 mm	StoneBreaker	46,1 ± 8,1	01:50 ± 00:28 min.:s
				EKL	79,4 ± 12,8	02:49 ± 00:37 min.:s
				Ho:YAG-Laser	563,6 ± 93,4	05:12 ± 00:58 min.:s
			Salivary stones	StoneBreaker	72,0 ± 14,6	02.42 ± 00:36 min.:s
				EKL	64,0 ± 25,2	02:59 ± 01:31 min.:s
				Ho:YAG-Laser	459,6 ± 349,4	02:52 ± 01:42 min.:s
