# Supplementary material for: Intracorporeal lithotripsy of salivary stones: in vitro comparison of different methods
Source: Eur Arch Otorhinolaryngol. 2025 Mar 7;282(6):3233–44. doi: 10.1007/s00405-025-09268-1 (PMC12122608; doi:10.1007/s00405-025-09268-1)
Supplement: Supplementary file 2 — Supplementary file2 (PDF 224 kb) [file 405_2025_9268_MOESM2_ESM.pdf]

Supplementary Information

Intracorporeal lithotripsy of salivary stones: in vitro comparison of different methods

European Archives of Oto-Rhino-Laryngology

Schulze Cathrin[1], Thangavelu Kruthika[1,], Gehrt Francesca[1], Schatton Robert[3], Keil Christian[2], Heers Hendrik[2], Abozenah Nermin H.[1], Stuck Boris A.[1], Geisthoff Urban[1]

[1] Klink für Hals-Nasen-Ohren Heilkunde, Kopf- und Hals-Chirurgie, Universitätsklinikum Marburg
[2] Klinik für Urologie Universitätsklinikum Marburg, Philipps-Universität Marburg,
[3] HNO Praxis Wülfrath

Address for correspondence:
Cathrin Schulze: Univ.-HNO-Klinik, Baldingerstrasse, 35043 Marburg, Tel. 06421 58-66488, Fax -66367,
Email: (cathrinschulze@gmx.de)

Online Resource 2

Table 3: Published in vivo studies regarding efficacy

Author	Year	Device	Stone	Number of treated stones and Success rate (%)	Treatment time mean
Gundlach	1990	Ho:YAG-Laser	Salivary stone	11/12 (92%)	-
Königsberger	1993	EHL	Salivary stone	20/29 (69%)	
Ito	1996	Ho:YAG-Laser (Pulsed-dye Laser)	Salivary stone	15/15 (100%)	30 min
Menezes	2000	EKL (Lithoclast)	Ureteric stones	19/22 (86%)	50 min
Keeley	2001	EKL	Ureteric stones	147/148 (99%)	-
Marchal	2002	EHL (Ho:YAG-Laser)	Salivary stone	90/110 (82%)	71 min
De Sior	2004	EKL (Lithoclast)	Ureteral stones	15/19 (78,9%)	55 min
Raif	2006	Ho:YAG-Laser (Er:YAG Laser)	Salivary stone	15/18 (83%)	-
Nakayama	2007	EHL	Salivary stone	1/1	-
Modayil	2008	EKL Swiss LithoClast (pneumatisch)	Salivary stone	1/1	45 min
Yu	2008	EHL	Salivary stone	17/21 (80,9%)	-
Nahlieli	2010	EHL	Salivary stone	-	-
Koch	2016	StoneBreaker	Salivary stone	48/49 (97,7%)	51 min
Serbetci	2010	Pneumatic lithotripter (Ho:YAG-Laser)	Salivary stone	-	-
Durbec	2012	Ho:YAG-Laser Thulium:YAG Laser	Salivary stone	37/40 (92%)	69 min
Martellucci	2013	Ho:YAG-Laser	Salivary stone	13/16 (81%)	-
Philips	2014	Ho:YAG-Laser	Salivary stone	13/16 (81%)	142,75 min
Sionis	2014	Ho:YAG Laser	Salivary stone	14/15 (93%)	45 min
Serbetci	2017	Pneumatic lithotripter	Salivary stone	30/34 (88%)	-
Koch	2019	Ho:YAG Laser	Salivary stone	12/12 and 75/75 (both 100%)	70,5 min and 106 min
Kaluzny	2020	Ho:YAG-Laser	Salivary stone	28/31 (90%)	49 min
Koch	2022	Pneumatic lithotripter Vibrolith	Salivary stone	76/77 (99%)	50 min

Table 4: published success rates and treatment times with PL, EKL, EHL and LL in in vivo studies

**Device**	**Success rate mean**	**Treatment time Mean**
PL	95,23%	48 min.
EKL	90,98%	50 min
EHL	82,98%	71 min.
LL	91,2 %	73,18 min

Table 5: published in vivo studies using PL (success rates and treatment times)

**Author**	**Year**	**Device**	**Stone**	**Number of treated stones and Success rate (%)**	**Treatment time mean**
Modayil	2008	EKL Swiss LithoClast (pneumatisch)	Salivary stone	1/1	45 min
Koch	2016	StoneBreaker	Salivary stone	48/49 (97,7%)	51 min
Serbetci	2010	Pneumatic lithotripter (Ho:YAG-Laser)	Salivary stone	-	-
Serbetci	2017	Pneumatic lithotripter	Salivary stone	30/34 (88%)	-
			mean	95,23%	48 min.

Table 6: published in vivo studies using EKL (success rates and treatment times)

**Author**	**Year**	**Device**	**Stone**	**Number of treated stones and Success rate (%)**	**Treatment time mean**
Menezes	2000	EKL (Lithoclast)	Ureteric stones	19/22 (86%)	50 min
Keeley	2001	EKL	Ureteric stones	147/148 (99%)	-
De Sior	2004	EKL (Lithoclast)	Ureteral stones	15/19 (78,9%)	55 min
Modayil	2008	EKL Swiss LithoClast (pneumatisch)	Salivary stone	1/1	45 min
			mean	90,98%	50 min

Table 7: published in vivo studies using EHL (success rates and treatment times)

Author	Year	Device	Stone	Number of treated stones and Success rate (%)	Treatment time mean
Königsberger	1993	EHL	Salivary stone	20/29 (69%)	
Marchal	2002	EHL (Ho:YAG-Laser)	Salivary stone	90/110 (82%)	71 min
Nakayama	2007	EHL	Salivary stone	1/1	-
Yu	2008	EHL	Salivary stone	17/21 (80,9%)	-
Nahlieli	2010	EHL	Salivary stone	-	-
			mean	82,98%	71 min.

Table 8: published in vivo studies using LL (success rates and treatment times)

Author	Year	Device	Stone	Number of treated stones and Success rate (%)	Treatment time mean
Gundlach	1990	Ho:YAG-Laser	Salivary stone	11/12 (92%)	-
Ito	1996	Ho:YAG-Laser (Pulsed-dye Laser)	Salivary stone	15/15 (100%)	30 min
Raif	2006	Ho:YAG-Laser (Er:YAG Laser)	Salivary stone	15/18 (83%)	-
Durbec	2012	Ho:YAG-Laser Thulium:YAG Laser	Salivary stone	37/40 (92%)	69 min
Martellucci	2013	Ho:YAG-Laser	Salivary stone	13/16 (81%)	-
Philips	2014	Ho:YAG-Laser	Salivary stone	13/16 (81%)	142,75 min
Sionis	2014	Ho:YAG Laser	Salivary stone	14/15 (93%)	45 min
Koch	2019	Ho:YAG Laser	Salivary stone	12/12 and 75/75 (both 100%)	70,5 min and 106 min
Kaluzny	2020	Ho:YAG-Laser	Salivary stone	28/31 (90%)	49 min
			mean	91,2 %	73,18 min
