# Supplementary material for: Intracorporeal lithotripsy of salivary stones: in vitro comparison of different methods
Source: Eur Arch Otorhinolaryngol. 2025 Mar 7;282(6):3233–44. doi: 10.1007/s00405-025-09268-1 (PMC12122608; doi:10.1007/s00405-025-09268-1)
Supplement: Supplementary file 3 — Supplementary file3 (PDF 205 kb) [file 405_2025_9268_MOESM3_ESM.pdf]

Supplementary Information

Intracorporeal lithotripsy of salivary stones: in vitro comparison of different methods

European Archives of Oto-Rhino-Laryngology

Schulze Cathrin[1], Thangavelu Kruthika[1,], Gehrt Francesca[1], Schatton Robert[3], Keil Christian[2], Heers Hendrik[2], Abozenah Nermin H.[1], Stuck Boris A.[1], Geisthoff Urban[1]

[1] Klink für Hals-Nasen-Ohren Heilkunde, Kopf- und Hals-Chirurgie, Universitätsklinikum Marburg
[2] Klinik für Urologie Universitätsklinikum Marburg, Philipps-Universität Marburg,
[3] HNO Praxis Wülfrath

Address for correspondence:
Cathrin Schulze: Univ.-HNO-Klinik, Baldingerstrasse, 35043 Marburg, Tel. 06421 58-66488, Fax -66367, Email: (cathrinschulze@gmx.de)

Online Resource 3

Table 9: Published in vitro studies regarding propulsion

**Author**	**Year**	**Setting**	**Stone phantom**	**Device**	**Propulsion**
Vorreuther	1998	Under water fragmentation and measurement of displacement	Chalk hard clay cubes	Lithoclast (pneumatic)	10 ± 5 cm
				EKL Combilith	5 ± 3 cm
Marguet	2005	Propulsion in horizontal tube	Cylindrical BegoStones	Lithoclast (pneumatic)	at 200 kPa: 9,9 mm
				Nd:YAG Laser	160 mJ, 5 Hz:7,6 cm<br>160 mJ, 10 Hz: 8,1 cm<br>160 mJ, 15 Hz: 6,8 cm
				Ho:YAG-Laser	0,8 J, 5 Hz: 3,3 cm<br>0,8 J, 10 Hz: 4,9 cm
Rühm	2014	Under water pendulum propulsion	Steel balls	LithoBreaker	Induced momentum<br>10^{-4}(kg m)/s
				StoneBreaker	Induced momentum<br>10^{-4}(kg m)/s
				Ho:YAG-Laser	Induced momentum<br>10^{-6} (kg m)/s
		Propulsion in horizontal tube	Steel balls	LithoBreaker	21,5 ± 2,7 cm
				StoneBreaker	20,3 ± 3,0 cm
Eisel	2018	Upward movement in vertical syringe	BegoStones	Ho:YAG-Laser	Long pulses 120,0 ± 2,9 mmxs^{-1} respectively 128,3 ± 6,9 mmxs^{-1}
Andreeva	2020	Horizontal tube	COM stones<br>Uric acid stones	Thulium fiber laser	1 mm
				Ho:YAG-Laser	Short pulse 3 mm<br>Long pulse 2 mm
This study		Horizontal gelatin tube<br>´	BegoStones	StoneBreaker	3,5 ± 0,7 cm
				EKL	0,9 ± 0,5 cm
				Ho:YAG-Laser	0,0 ± 0,0 cm

Table 10: Published in-vitro studies regarding propulsion – PL only

**Author**	**Year**	**Setting**	**Stone phantom**	**Device**	**Propulsion**
Vorreuther	1998	Under water fragmentation and measurement of displacement	Chalk hard clay cubes	Lithoclast (pneumatic)	10 ± 5 cm
Marguet	2005	Propulsion in horizontal tube	Cylindrical BegoStones	Lithoclast (pneumatic)	at 200 kPa: 9,9 mm
Rühm	2014	Under water pendulum propulsion	Steel balls	StoneBreaker	Induced momentum 10^{-4}(kg m)/s
		Propulsion in horizontal tube	Steel balls	StoneBreaker	20,3 ± 3,0 cm
				mean	15,15 cm

Table 11: Published in-vitro studies regarding propulsion – EKL only

**Author**	**Year**	**Setting**	**Stone phantom**	**Device**	**Propulsion**
Vorreuther	1998	Under measurement of displacement	Chalk hard clay cubes	EKL Combilith	5 ± 3 cm
				mean	5 cm

Table 12: Published in-vitro studies regarding propulsion – LL only

**Author**	**Year**	**Setting**	**Stone phantom**	**Device**	**Propulsion**
Marguet	2005	Propulsion in horizontal tube	Cylindrical BegoStones	Ho:YAG-Laser	0,8 J, 5 Hz: 3,3 cm<br>0,8 J, 10 Hz: 4,9 cm
Rühm	2014	Under water pendulum propulsion	Steel balls	Ho:YAG-Laser	Induced momentum 10^{-6} (kg m)/s
Eisel	2018	Upward movement in vertical syringe	BegoStones	Ho:YAG-Laser	Long pulses 120,0 ± 2,9 $mmxs^{-1}$ respectively 128,3 ± 6,9 $mmxs^{-1}$
Andreeva	2020	Horizontal tube	COM stones<br>Uric acid stones	Ho:YAG-Laser	Short pulse 3 mm<br>Long pulse 2 mm
